# Book Reviews

**Published:** 1899-12

**Authors:** 


### *BOOK REVIEWS, PAMPHLETS, EXCHANGES.*

### *BOOK REVIEWS.*

**[Contributions solicited for review.]**

A Manual of Diseases of the Nervous System. By Sir W. R. Gowers, M.D., F.R.C.P., F.R.S., Consulting Physician to University College Hospital; Physician to the National Hospital for the Paralyzed and Epileptic, Queen Square. Third edition, revised and enlarged. Edited by Sir W. R. Gowers and James Taylor , M.A., M.D., F.R.C.P., Senior Assistant Physician to the National Hospital for the Paralyzed and Epileptic, Queen Square; Physician to the Northeastern Hospital for Children and to the National Orthopædic Hospital. Volume I., Diseases of the Nerves and Spinal Cord. With 192 illustrations. Octavo. Price, net, $4.00. P. Blakiston's Son & Co., 1012 Walnut street, Philadelphia.

Gowers's text-book has held its place at the head of nerve works ever since its first publication. There is no other treatise in the English language approaching it in excellence. The present edition, of which the first volume is published, has been carefully revised by Dr. Taylor and contains over 75 pages of new matter. We can only speak in the highest terms of the treatment accorded the subject-matter.

---

A System of Medicine. By Many Writers. Edited by Thomas Clifford Allbutt, M.A., M.D., LL.D., F.R.S., etc. Published by Macmillan & Co. Pp. 937. Price $5.00.

This System was expected, at its inception, to end with the 6th volume, but as time passed it was found necessary to add two more. The present is the second last of the series. In it diseases of the nervous system are continued, but not concluded, for volume VIII. will deal fully with mental diseases as well as diseases of the skin. The present volume takes up diffuse and limited diseases of the spinal cord, nuclear diseases and diseases of the brain, including experimental pathology, and regional diagnosis. To these are added diseases of sleep, puerperal eclampsia, chorea, the tics, etc. Among the contributors are such famous names as Fred Taylor, Risien Russell, Batten, Grainger Stewart, Ormond, Mott, Beevor, Starr, Ferrier, Hill, Bastian, Barlow, Colman, Bennett, Ballance, Tooth, Bramwell, Turner, Bulloch, Goodall, Kingdon, Gowers, Herman, and Bradbury. The volume contains some excellent illustrations, and is on the high level of those which have preceded it.

---

PATHOLOGY AND TREATMENT OF SEXUAL IMPOTENCE. By Victor G. Vecki, M.D., from the second German edition, revised and rewritten. W. B. Saunders, Philadelphia, 1899. Price, $2 net.

Vecki's work on Sexual Impotence is entitled to careful reading and consideration. The subject is of the highest importance in spite of the tendency in the medical profession to taboo it. The various types of impotence are considered, with chapters on the physiology, anatomy and pathology of the male sexual organs. The recommendations for treatment are good, but the author's attitude toward drug therapy, as applied to sexual impotence, is rather pessimistic. He gives something of an indorsement to Gassen's apparatus for mechanical relief of the difficulty. The book is well written; here and there the word-painting of sexual matters is rather too vivid. Those interested in genito-urinary diseases would do well to purchase this book.

---

A LABORATORY MANUAL OF PHYSIOLOGICAL CHEMISTRY. By Elbert W. Rockwood, B.S., M.D., Professor of Chemistry and Toxicology in the University of Iowa. Illustrated with one colored plate and three plates of Microscopic Preparations, 5⅜x7⅝ inches. Pages viii–204. Extra cloth, $1.00, net, The F. A. Davis Co., Publishers, 1914–16 Cherry street, Philadelphia.

This is an exceedingly valuable little book for the practical worker in physiological chemistry. Its author has given in a concise form almost everything that is embraced under the subject. The style is clear and the arrangement most excellent. Blank sheets can be found throughout the book affording a place where the student can "jot down notes." Physiological Chemistry deserves a more extended study by the medical student and practitioner alike than has heretofore been done, and we predict for this little book a well-deserved place. The binding is exceedingly attractive.

---

THE GROSS AND MINUTE ANATOMY OF THE NERVOUS SYSTEM. By H. C. Gordinier, A.M., M.D., Professor of Physiology and of the Anatomy of the Nervous System in the Albany Medical College; Member American Neurological Association. With 48 full-page plates and 213 other illustrations, many of which are printed in colors, a large number being from original sources. Octavo. Price, net, $6.00 Cloth; $7.00 Sheep. P. Blakiston's Son & Co., 1012 Walnut street, Philadelphia, Pa.

A very clear and comprehensive work written for student and practitioner, with chapters on Cerebral Localization, Embryology and methods of staining. Not the least interesting page is a history of a case of agraphia due to a lesion in the second left frontal convolution that has a direct bearing on the mooted point of a special writing center.

---

MINERAL WATERS OF THE UNITED STATES AND THEIR THERAPEUTIC USES. By James K. Crook, M.D., New York. Lea Bros. & Co., New York and Philadelphia, 1899.

This book will prove a guide to the physician in selecting suitable mineral springs to which he desires to send his patients, and will furnish all the information desired as to the chemical constituents of the waters, hotel accommodations, railroads, and the nature of the country where the various springs are situated. The book contains a description of all the mineral springs and health resorts of the United States, and the descriptions are sufficiently full to cover all points likely to be inquired into in making a selection.

---

ATLAS OF DISEASES OF THE SKIN, INCLUDING AN EPITÔME OF PATHOLOGY AND TREATMENT. By Professor Dr. Franz Mracék of Vienna. Edited by Henry W. Stelwagon, M.D., Ph.D., Philadelphia, with 63 colored plates and 39 full-page half-tone illustrations. Philadelphia. W. B. Saunders, 1899. Price $2.50.

This manual commends itself for the excellence of its illustrations. It may very well take the place of the very expensive atlases of skin diseases on the market, and is commendable alike to the general practitioner and the specialist in this province. Many of the illustrations are faithful reproductions of nature, showing the color effects of cutaneous lesions; others are half-toned reproductions of photographs. The price of the work is $2.50, and is well worth it.

---

ESSENTIALS OF MEDICAL CHEMISTRY, ORGANIC AND INORGANIC. By Lawrence Wolff, M.D., Demonstrator of Chemistry in Jefferson Medical College, Philadelphia. Fifth edition revised by Smith E. Jelliffe, New York College of Pharmacy. W. B. Saunders, Philadelphia, 1899. Price $1.

This work contains 222 pages and is the fifth edition. It is devoted to medical chemistry, toxicology, medical physics and analyses of various excretions and secretions. It is arranged in the form of questions and answers which has proved so popular with medical students, and is a very attractive feature of Saunders's Question Compends.

LEA'S POCKET TEXT-BOOK SERIALS up to date comprise Malsbary's Practice of Medicine; Diseases of Children, by Geo. M. Tuttle of St. Louis; Materia Medica, Therapeutics, Medical Pharmacy, Prescription Writing and Medical Latin, by Wm. Schleif, Ph.G., M.D., Instructor in Pharmacy in the University of Pennsylvania; A Manual of Physiology, by Howard D. Collins and W. H. Rockwell, of the College of Physicians and Surgeons, New York. These books are uniform in size and binding, and give the essentials of the subject considered in a manner suitable for the student of medicine. The books are bound in red with gilt illuminated backs, and are the most ornate of any series of manuals we have yet seen.

